# Evaluation of Turkish Women’s Thoughts on Their First Sexual Experience

**DOI:** 10.7759/cureus.14096

**Published:** 2021-03-24

**Authors:** Gokcen Erdogan

**Affiliations:** 1 Gynecology and Obstetrics, Near East University Medical Faculty, Nicosia, CYP

**Keywords:** sexuality, first sexual experience, turkish women

## Abstract

Objective

This study aims to determine the need for sexual health education, whose importance is also emphasized by the World Health Organization, so that the youths can develop a responsible, healthy, and satisfactory sexual life and evaluates Turkish women’s thoughts about their first sexual experience.

Methods

A total of 100 persons who were willing to participate in the research, selected through random sampling from among the women above 18 years of age who have had their first sexual experiences, and applied to the Private Diva Gynecological Diseases and Women's Health Clinic in Cankaya district of Ankara province for gynecological examination, hymen examination, or pregnancy diagnosis from 01/06/2020 to 01/09/2020.

Results

Of the participants, 63% were undergraduate students and 47% had stated that their first sexual experience is a private experience, while 42% stated that they were not careful about protection during their first sexual experience. It is worth noting that those who were careful about protection were the participants in the age range of 18-25 (45.5%). It was seen that 80% of those who live in the Black Sea Region have not had any sexual activity before their first sexual experience and that 46.7% of participants who live in this region again were not careful about protection.

Conclusion

In the link from healthy individuals to a healthy society, sexual education must be started before their first sexual experience and continued throughout life.

## Introduction

Sexual behaviors and experiences are the sexual behaviors and activities the individuals carry out continuously on their own or together with others, in the form of a habit and as an expression of their sexuality. Since sexuality refers to a multidimensional and combined experience, the points of view on sexuality are rather diversified. Today, it is known that numerous fears related to sexuality, the anxieties about pain, bleeding, strain regarding the first night stem from social myths. In today's society, although sexuality is still taboo and forbidden to talk about from certain perspectives and in certain geographical regions, a certain social segment has adopted a modern point of view, and now sexuality is being discussed within the scope of needs and human rights [1-5]. 

Since sexuality is an important part of human life and general health, while experiencing it through a healthy pattern affects the individual, family, and society positively, the impairment of sexual health affects the family and society negatively by impairing the individual's physical and psychological health. The protection, improvement, and sustainability of sexual health is possible only by accessing sufficient and correct information, i.e., sexual health education. Family is the institution where the initial sexual information arises and is formed, and it is known that Turkish society has a traditional and conservative structure regarding sexual education, and that talking about sexuality is considered shameful and sinful, and thus forbidden. The children and teenagers who can receive the information they need in neither their families nor systemic and organized education try to obtain such information through informal means, and this effort can result in obtaining harmful, incorrect, or incomplete information, and thus having negative experiences. Among these negative experiences, very grave examples such as premature sexual experiences, addiction to pornography, misogyny, unwanted pregnancies, premature marriages, deliveries and abortions in unhealthy and unsafe environments, sexual function disorders, and maternal and infant mortality can be mentioned [6].

When a comprehensive sexual education is planned in compliance with the individual's age, developmental period, and cultural structure, it enables the children and youths to develop behavioral patterns such as displaying healthy physical and emotional development, having positive body image, displaying conscious and logical sexual behaviors, having positive feelings about their own genders, being able to express themselves and their problems regarding sexual matters, telling the sexual behaviors apart as positive and negative ones and protecting themselves from unintended pregnancies, harassment and abuse, and the sexually transmitted diseases [7].

The objective of this study is to learn Turkish women's thoughts about their first sexual experience, determine the educational needs of different groups, and contribute to the body of literature on a subject that is considered culturally taboo and discussed scarcely.

## Materials and methods

A total of 100 persons who were willing to participate in the research, selected through random sampling from among the women above 18 years of age who have had their first sexual experiences, and applied to the Private Diva Gynecological Diseases and Women's Health Clinic in Cankaya district of Ankara province for gynecological examination, hymen examination, or pregnancy diagnosis from 01.06.2020 to 01.09.2020. Ethics committee permission was obtained before starting the study. The study was conducted in accordance with the ethical principles of the Declaration of Helsinki.

Two different data collection forms have been used in this research.

1. Socio-demographic data collection form

The questionnaire prepared to collect information on the participants' age range, geographical regions of origin, educational backgrounds, occupations, marital status, and whether they have an active sexual life.

2. Questionnaire for the evaluation of thoughts and behaviors regarding the first sexual experience

The questionnaire was prepared by the researcher to understand the thoughts and behavioral patterns regarding the first sexual experience that results in the breaking of the hymen, which is a conspicuous and social problem according to a review of the relevant scientific literature by the researcher. The questionnaire comprised eight statements and allowed giving replies on a 3-point Likert scale (agree, neither agree nor disagree, disagree).

The following statements were included in this questionnaire:

· I used to have sexual activities before my first sexual experience as well.

· My first sexual partner was someone I had an emotional bond with.

· I had my first sexual experience willingly.

· I'm not against premarital sex.

· My first sexual experience was a planned one.

· I was careful about protection during my first sexual experience.

· My first sexual experience is special to me.

· I had expectations from my first sexual experience.

Statistical analysis

The data have been analyzed through SPSS 23 software package (IBM Corp, Armonk, NY, USA). While evaluating the study data, the frequency distributions (number, percentage) and the descriptive statistics (average, standard deviation) have been used for the categorical variables and the quantitative variables respectively. The findings have been presented, interpreted, and discussed in tables. In the evaluation of the results, p ≤ 0.05 has been accepted as statistically significant.

## Results

The mean age of the 100 individuals participating in the study was 24.57 ± 3.88 years: 66 participants in the 18-15 years age group and 34 participants in the 26-35 years age range. Educational levels were determined as university and above in 72 participants, high school in 22 participants, and primary education in six participants. While 58 of the participants stated that they lived in the Central Anatolian region, 15 of them stated that they lived in the Black Sea region, 11 them stated that they lived in the Marmara region, and 16 them stated that they lived in other regions. Forty-two of the participants were students and 58 participants worked. All of them were single and 20 of them had an active sex life.

**Figure 1:**
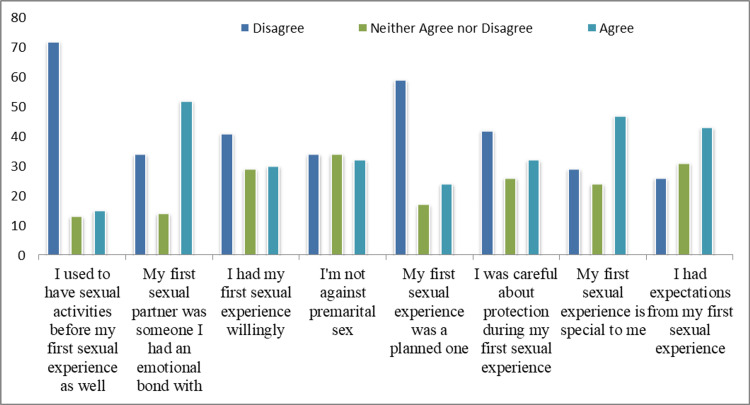
Responses of the participants on their first sexual experience

The answers of the participants regarding their first sexual experience are provided in Figure 1.

**Figure 2:**
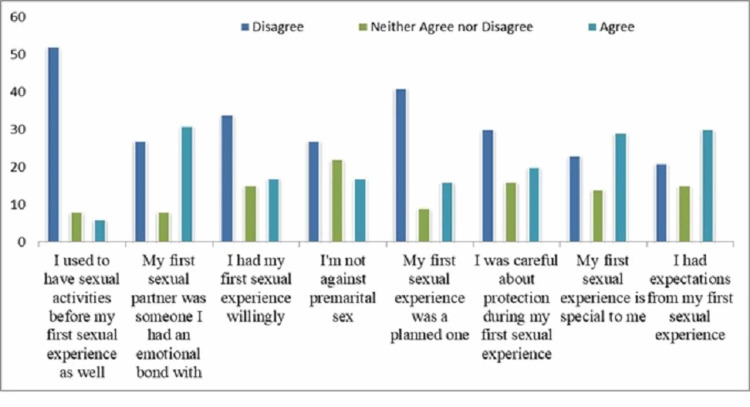
Responses of participants in the age group 18-25 years on their first sexual experience

**Figure 3:**
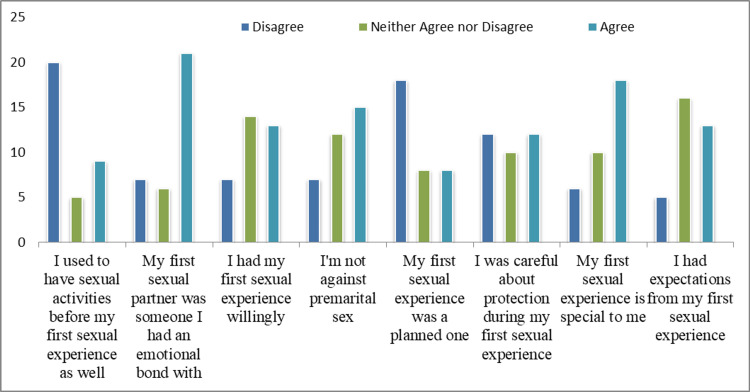
Responses of participants in the age group 26-35 years on their first sexual experience

The answers of the participants in the age group 18-25 years to the statements about their first sexual experience are given in Figure 2 and the answers of the participants in the age group 26-35 years are given in Figure 3.

The distribution of the participants according to their educational background in relation to the answers they gave to the statements regarding the first sexual experience is given in Table 1.

**Table 1:** Distribution of participants by the relation between educational background and replies to statements on first sexual experience

		High School Degree and Below		Associate's Degree and Above	
		n	%	n	%
I used to have sexual activities before my first sexual experience as well	Disagree	18	64.3	54	75.0
	Neither Agree nor Disagree	4	14.3	9	12.5
	Agree	6	21.4	9	12.5
My first sexual partner was someone I had an emotional bond with	Disagree	12	42.9	22	30.6
	Neither Agree nor Disagree	5	17.9	9	12.5
	Agree	11	39.3	41	56.9
I had my first sexual experience willingly	Disagree	13	46.4	28	38.9
	Neither Agree nor Disagree	6	21.4	23	31.9
	Agree	9	32.1	21	29.2
I'm not against premarital sex	Disagree	12	42.9	22	30.6
	Neither Agree nor Disagree	6	21.4	28	38.9
	Agree	10	35.7	22	30.6
My first sexual experience was a planned one	Disagree	18	64.3	41	56.9
	Neither Agree nor Disagree	5	17.9	12	16.7
	Agree	5	17.9	19	26.4
I was careful about protection during my first sexual experience	Disagree	15	53.6	27	37.5
	Neither Agree nor Disagree	7	25.0	19	26.4
	Agree	6	21.4	26	36.1
My first sexual experience is special to me	Disagree	8	28.6	21	29.2
	Neither Agree nor Disagree	10	35.7	14	19.4
	Agree	10	35.7	37	51.4
I had expectations from my first sexual experience	Disagree	10	35.7	16	22.2
	Neither Agree nor Disagree	10	35.7	21	29.2
	Agree	8	28.6	35	48.6

The distribution of the participants by the relationship between the geographical regions and the answers given to the statements about the first sexual experience is provided in Table 2.

**Table 2:** Distribution of participants by relationship between geographical regions and answers to statements on first sexual experience

		Mediterranean/ Aegean/ Marmara		Central Anatolia		Black Sea		Eastern/ Southeastern Anatolia	
		n	%	n	%	n	%	n	%
I used to have sexual activities before my first sexual experience as well	Disagree	11	57.9	44	75.9	12	80.0	5	62.5
	Neither Agree nor Disagree	3	158	8	13.8	2	13.3	0	0.0
	Agree	5	26.3	6	10.3	1	6.7	3	37.5
My first sexual partner was someone I had an emotional bond with	Disagree	3	15.8	22	37.9	5	33.3	4	50.0
	Neither Agree nor Disagree	2	10.5	8	13.8	3	20.0	1	12.5
	Agree	14	73.7	28	48.3	7	46.7	3	37.5
I had my first sexual experience willingly	Disagree	3	15.8	27	46.6	7	46.7	4	50.0
	Neither Agree nor Disagree	6	31.6	17	29.3	5	33.3	1	12.5
	Agree	10	52.6	14	24.1	3	20.0	3	37.5
I'm not against premarital sex	Disagree	2	10.5	24	41.4	5	33.3	3	37.5
	Neither Agree nor Disagree	8	42.1	19	32.8	4	26.7	3	37.5
	Agree	9	47.4	15	25.9	6	40.0	2	25.0
My first sexual experience was a planned one	Disagree	7	36.8	37	63.8	9	60.0	6	75.0
	Neither Agree nor Disagree	2	10.5	9	15.5	5	33.3	1	12.5
	Agree	10	52.6	12	20.7	1	6.7	1	12.5
I was careful about protection during my first sexual experience	Disagree	4	21.1	26	44.8	7	46.7	5	62.5
	Neither Agree nor Disagree	4	21.1	18	31.0	3	20.0	1	12.5
	Agree	11	57.9	14	24.1	5	33.3	2	25.0
My first sexual experience is special to me	Disagree	3	15.8	19	32.8	4	26.7	3	37.5
	Neither Agree nor Disagree	4	21.1	12	20.7	6	40.0	2	25.0
	Agree	12	632	27	46.6	5	33.3	3	37.5
I had expectations from my first sexual experience	Disagree	3	15.8	15	25.9	5	33.3	3	37.5
	Neither Agree nor Disagree	7	36.8	18	31.0	4	26.7	2	25.0
	Agree	9	47.4	25	43.1	6	40.0	3	37.5

The distribution of the participants according to the answers given to the statements related to professional status and first sexual experience is seen in Table 3.

**Table 3:** Distribution of participants by occupation and their replies to expressions on first sexual experience

		Clerk		Student		Others (Self-Employed, Sales Representative, Worker, Housewife, etc.)	
		n	%	n	%	n	%
I used to have sexual activities before my first sexual experience as well	Disagree	15	62.5	34	81.0	23	67.6
	Neither Agree nor Disagree	4	16.7	5	11.9	4	11.8
	Agree	5	20.8	3	7.1	7	20.6
My first sexual partner was someone I had an emotional bond with	Disagree	4	16.7	17	40.5	13	38.2
	Neither Agree nor Disagree	7	29.2	6	14. 3	1	2.9
	Agree	13	54.2	19	45.2	20	58.8
I had my first sexual experience willingly	Disagree	8	33.3	22	52.4	11	32.4
	Neither Agree nor Disagree	11	45.8	10	23.8	8	23.5
	Agree	5	20.8	10	23.8	15	44.1
I'm not against premarital sex	Disagree	10	41.7	16	38.1	8	23.5
	Neither Agree nor Disagree	8	33.3	13	31.0	13	38.2
	Agree	6	25.0	13	31.0	13	38.2
My first sexual experience was a planned one	Disagree	15	62.5	25	59.5	19	55.9
	Neither Agree nor Disagree	5	20.8	7	16.7	5	14.7
	Agree	4	16.7	10	23.8	10	29.4
I was careful about protection during my first sexual experience	Disagree	11	45.8	15	35.7	16	47.1
	Neither Agree nor Disagree	7	29.2	14	33.3	5	14.7
	Agree	6	25.0	13	31.0	13	38.2
My first sexual experience is special to me	Disagree	7	29.2	15	35.7	7	20.6
	Neither Agree nor Disagree	5	20.8	8	19.0	11	32.4
	Agree	12	50.0	19	45.2	16	47.1
I had expectations from my first sexual experience	Disagree	5	20.8	14	33.3	7	20.6
	Neither Agree nor Disagree	8	33.3	8	19.0	15	44.1
	Agree	11	45.8	20	47.6	12	35.3

Table 4 shows the distribution of the participants according to their status of having an active sexual life and the answers given to the statements related to the first sexual experience.

**Table 4:** Distribution of participants by active sexual life and their replies to statements on first sexual experience

		Yes		No	
		n	%	n	%
I used to have sexual activities before my first sexual experience as well	Disagree	8	40.0	64	80.0
	Neither Agree nor Disagree	4	20.0	9	11.3
	Agree	8	40.0	7	8.8
My first sexual partner was someone I had an emotional bond with	Disagree	2	10.0	32	40.0
	Neither Agree nor Disagree	2	10.0	12	15.0
	Agree	16	80.0	36	45.0
I had my first sexual experience willingly	Disagree	3	15.0	38	47.5
	Neither Agree nor Disagree	4	20.0	25	31.3
	Agree	13	65.0	17	21.3
I'm not against premarital sex	Disagree	0	0	34	42.5
	Neither Agree nor Disagree	5	25.0	29	36.3
	Agree	15	75.0	17	21.3
My first sexual experience was a planned one	Disagree	4	20.0	55	68.8
	Neither Agree nor Disagree	5	25.0	12	15.0
	Agree	11	55.0	13	16.3
I was careful about protection during my first sexual experience	Disagree	4	20.0	38	47.5
	Neither Agree nor Disagree	4	20.0	22	27.5
	Agree	12	60.0	20	25.0
My first sexual experience is special to me	Disagree	3	15.0	26	32.5
	Neither Agree nor Disagree	3	15.0	21	26.3
	Agree	14	70.0	33	41.3
I had expectations from my first sexual experience	Disagree	2	10.0	24	30.0
	Neither Agree nor Disagree	9	45.0	22	27.5
	Agree	9	45.0	34	42.5

The data indicates that the rate of those who are sexually active is 12.1% for the age range of 16-25 years and 35.3% for the age range of 26-35 years. In consequence of the chi-square analysis made, there is a statistically significant relationship between age and sexual activity (p≤0.05). Thus, the rate of those who are sexually active in the age range of 26-35 years is significantly higher than those in the age range of 16-25 years.

The distribution of the participants according to the relationship between pre-marital sexual experience thinking and protection in the first sexual experience is shown in Table 5.

**Table 5:** Distribution of participants by relationship between their thoughts on premarital sex and protection in first sexual intercourse The indices a and b have been used to indicate differences. There is a difference between the rates that include different letters. *significant at p ≤ 0.05.

			I'm not against Premarital Sex			Total	Chi-Square	p
			Disagree	Neither Agree nor Disagree	Agree			
I was careful about Protection during My First Sexual Experience	Disagree	Number	21	10	11	42	22.742	0. 000*
		Percentage	61.8_a_	29.4_b_	34.4_a,b_	42.0		
	Neither Agree nor Disagree	Number	12	10	4	26		
		Percentage	35.3_a_	29.4_a_	12.5_a_	26.0		
	Agree	Number	1	14	17	32		
		Percentage	2.9_a_	41.2_b_	53.1_b_	32.0		
Total		Number	34	34	32	100		
		Percentage	100.0	100.0	100.0	100.0		

## Discussion

Numerous factors that organize the functions such as the individual's biological and psychological development and interpersonal relations also have a role in the commencement or continuation of sexual function disorders [8]. As is the case in numerous countries, sexuality is a difficult subject to talk about in Turkey due to numerous reasons such as social, cultural, communal, and religious ones. While evaluating the individuals' sexual functions, the evaluator learns their psychosocial, developmental, and sexual histories, and tries to access the subjects in those histories, such as the current sexual life, emotional health, relationship capacity, past experiences, development of gender identity, the first sexual experience, and traumas [9].

In their study, Ozkan and Beji evaluated the effects of the psychological and interpersonal factors on sexual function and emphasized that numerous women's emotional and behavioral reactions to their first sexual experiences were fear, anxiety, and conflict, depending on the value judgments of the society in which they live and the gender roles imposed on women by that society, and that extramarital affair is taboo in numerous societies [10]. Of the participants of this study, 47% said, “My first sexual experience is special to me,” and 52% said, “My first sexual partner was someone I had an emotional bond with.”

In their studies, Senoglu, Coban, and Karacam emphasize that the pregnancies caused by extramarital sexual experiences can result in women paying a heavy cost [11]. Besides, the rate of unwanted pregnancies and willful miscarriages, which we encounter as a serious women's health problem in consequence of this experience, is 42% in Turkey. Besides, according to the Turkey Demographic and Health Survey Report 2013, 5% of adolescent women have given birth to children. The reasons for adolescent pregnancies and unintended pregnancies include inability in accessing contraceptive methods, failure in using them effectively and correctly, or not using the contraceptive methods at all. Society's negative and judgmental look at pregnancies caused by extramarital affairs prevents these women from receiving service from healthcare centers and filling their knowledge gaps. Women have been forced to pay a heavy cost due to their pregnancies caused by extramarital affairs. These costs can range from having a miscarriage by using traditional methods to having an abortion under inappropriate conditions, and even death. In this study, it is seen that only 2.9% of the participants who said “I'm not against premarital sex” in Table 4 also said, “I was careful about protection during my first sexual experience”. Whether those who said that they were not against premarital sex have had such an experience, on the other hand, could be the subject of another research.

At this point, it is suggested that the healthcare personnel who give information and service regarding the subjects such as sexually transmitted diseases, protection from unwanted pregnancies be trained, through on-the-job trainings at certain intervals, about the importance and dimensions of their job, given information on the sexuality matters, and enabled to offer the service they offer equally to all social segments without regard to individual differences such as marital status or age. 

One of the striking results of this study is the significant difference among the geographical regions. The rate of the women who live in the Black Sea region who said that they had not been careful about protection during their first sexual experience is 46.7%, while 57. 9% of the women who live in the Mediterranean/Aegean/Marmara region said that they had been careful about protection during their first sexual experience. At this point, it is suggested that the sexual health training to be planned should be enhanced so that it can meet the needs of the relevant geographical region as well as having special contents on the basis of the periodic features and educational background.

According to Bilgin and Kömürcü, women have a more comfortable and active sexual life with a regular partner in their adulthood period, and again in this period, they show changes regarding the social and sexual roles. They have stated that sexuality is a learnable behavior and a woman in her adulthood learns sexual behaviors and develops her ability to feel pleasure and reach orgasm [12].

Our study indicates that, in the age range of 26-35 years, those who said that they had an active sexual life are significantly higher, by 35.3%, than those in the age range of 18-25 years. Besides, our study indicates that 60% of those who had an active sexual life were careful about protection during their first sexual experiences, 65% had their first sexual experiences willingly, and 80% had their first sexual experiences with someone they were emotionally bound to. This may lead us to the conclusion that those who had their first sexual experiences willingly have been able to participate in adult sexual life actively. Besides, it is seen that 80% of those who said that they had had no sexual activity before their first sexual experience do not have active sexual life; and this shows the importance of starting the sexual education in an early period and determining its features on the basis of the periods of life.

However, whether having an active sexual life leads to displaying responsible behaviors or those who have developed responsible behaviors through education have an active sexual life and get protection against sexually transmitted diseases and unintended pregnancies, in other words, the question of which situation is the precursor of the other could be the subject of another research.

Another subject emphasized in the study of Bilgin and Kömürcü [12] is the fact that, regardless of having a sex partner, masturbation, self-satisfaction, and orgasm are important in women's getting to know sexuality, and that the women can describe sexuality as the activity performed with a partner or in various other different ways, including also masturbation. And that 15% of the women in this study said that they had had sexual activity before the first sexual experience while the fact that 72% said "no" indicates that alternative ways in getting to know sexuality may also be included in the education subject. In this way, women will be able to experience sexuality and get to know their own body and sexuality in alternative and safe ways even in an early period of their lives.

As is the case with numerous studies, the study of Bilgin and Kömürcü [12] also states sexual harassment and rape as the reason for women's sexual problems; and it has been found out that a minimum of 30-60% of women had faced sexual problem at least once in their lives.

Considering that this traumatic life started with the first sexual experience, it is possible to foresee that the potential results are more hurtful. Besides, it is emphasized that the sense of psychological commitment is more effective than estrogen and testosterone in the woman and her partner having sexual desire; and in this study, 41% of the women said that their first sexual experiences had not been a willing one [12]. However, although the experience was an unwilling one, under the influence of which factor it took place and whether there was any underlying story of harassment, rape, or abuse could be the subjects of another study.

When we look at Bahar’s study "Perception of Sexuality by Generation Y", the generation Y, as used by most experts for those who were born between the years 1980 and 2001 while there are also other different definitions for the generation Y, is the first generation that has grown up under the influence of computer and the internet and also the one that has the oldest parents compared to the other generations; and the parents of one-fourth of the generation Y got college education for at least once. Generation Y is a generation that has had the advantages such as fast access to information, advanced thinking, and analyzing competence. Naturally, the ways of education and information-gathering have changed for this generation, simultaneously with the change in the traditional family structure. The average age of those who have participated in this study is 24.57 years, and this plays a supportive role in reflecting the sexuality perception of generation Y, which was once called the Next Generation. In Bahar’s study, when the participants were asked questions about their first relationships and first sexual experiences, four out of 24 participants said that they generally disapproved of premarital sex, and that this was unacceptable for them and their families. 20 persons regarded it as a requirement of life and a normal situation. On the other hand, the rates of those who were against, those who are not against, and those who are indecisive about premarital sex were almost equal. Of the participants, 32% said “I'm not against premarital sex” while 34% were indecisive and 34% said that they were against it [13].

This result indicates the transition from traditional period to the internet period in information-gathering emphasizes the importance of conveying sexuality education to individuals through different access channels. In other words, when sexuality education is made available to individuals through different access channels like the internet, TV, newspapers, and organized education, it will also be possible to educate the individuals who are the representatives of the transition era.

The rate of those who agree with the expression “I used to have sexual activities before my first sexual experience as well” is significantly different for the students compared to employees, and this rate is 81%. Thus, it is possible to conclude that the most effective source to give information is organized education. It is suggested that the education given in the organized education institutions be given by the persons who are competent to give education on these subjects.

Limitations of the study

This study has some limitations. First of all, the study was run in a single center, and was carried out only under the conditions of the center where the study was conducted. The results of the study cannot be generalized. However, given the lack of studies in the literature on this issue, we believe that our results would provide a significant contribution.

## Conclusions

In conclusion, sexual health education is of great importance for the women in Turkish society to develop a positive attitude towards sexuality, create a healthy and satisfactory sexual life, develop responsible and safe behaviors, and willingly without any coercion, regard sexual health as part of the general health and natural activity. This education must be planned specifically for each age, period, region, and educational background, and offered equally to all by competent persons. In the link from healthy individuals to a healthy society, sexual education must be started before the first sexual experience and continued throughout life.
